# Touch and olfaction/taste differentiate children carrying a 16p11.2 deletion from children with ASD

**DOI:** 10.1186/s13229-020-00410-w

**Published:** 2021-02-05

**Authors:** Joana Maria Almeida Osório, Borja Rodríguez-Herreros, David Romascano, Vincent Junod, Aline Habegger, Aurélie Pain, Sonia Richetin, Paola Yu, Bertrand Isidor, Lionel Van Maldergem, Linda Pons, Sabine Manificat, Nadia Chabane, Marine Jequier Gygax, Anne Manuela Maillard

**Affiliations:** 1 CHUV-Centre Hospitalier Universitaire Vaudois, Service des Troubles du Spectre de l’Autisme et apparentés, Lausanne University Hospital, Les Allières – Av. Beaumont 23, 1011 Lausanne, Switzerland; 2 Laboratory for Investigative Neurophysiology (LINE), Department of Radiology, Lausanne University Hospital and University of Lausanne, Lausanne, Switzerland; 3 Service de Génétique Médicale, CHU-Nantes, Nantes, France; 4 Centre de Génétique Humaine, Centre Hospitalier Régional Universitaire, Université de Franche-Comté, Besançon, France; 5 Unité de recherche en neurosciences intégratives et cognitives EA481, Université de Franche-Comté, Besançon, France; 6 Centre d’investigation clinique 1431, INSERM, Besançon, France; 7 Service Génopsy - Pôle Hospitalo-Universitaire ADIS, Centre hospitalier Le Vinatier, Bron, France

**Keywords:** Autism spectrum disorder (ASD), 16p11.2 deletion, Copy number variants (CNV), Sensory processing, Touch, Olfaction, Children, Sensory processing measure (SPM)

## Abstract

**Background:**

Sensory processing atypicalities are frequent in Autism Spectrum Disorder (ASD) and neurodevelopmental disorders (NDD). Different domains of sensory processing appear to be differentially altered in these disorders. In this study, we explored the sensory profile of two clinical cohorts, in comparison with a sample of typically developing children.

**Methods:**

Behavioral responses to sensory stimuli were assessed using the Sensory Processing Measure (parent-report questionnaire). We included 121 ASD children, 17 carriers of the 16p11.2 deletion (Del 16p11.2) and 45 typically developing (TD) children. All participants were aged between 2 and 12 years. Additional measures included the Tactile Defensiveness and Discrimination Test-Revised, Wechsler Intelligence Scales and Autism Diagnostic Observation Schedule (ADOS-2). Statistical analyses included MANCOVA and regression analyses.

**Results:**

ASD children score significantly higher on all SPM subscales compared to TD. Del16p11.2 also scored higher than TD on all subscales except for tactile and olfactory/taste processing, in which they score similarly to TD. When assessing sensory modulation patterns (hyper-, hypo-responsiveness and seeking), ASD did not significantly differ from del16p11.2. Both groups had significantly higher scores across all patterns than the TD group. There was no significant association between the SPM Touch subscale and the TDDT-R.

**Limitations:**

Sensory processing was assessed using a parent-report questionnaire. Even though it captures observable behavior, a questionnaire does not assess sensory processing in all its complexity. The sample size of the genetic cohort and the small subset of ASD children with TDDT-R data render some of our results exploratory. Divergence between SPM Touch and TDDT-R raises important questions about the nature of the process that is assessed.

**Conclusions:**

Touch and olfaction/taste seem to be particularly affected in ASD children compared to del16p11.2. These results indicate that parent report measures can provide a useful perspective on behavioral expression. Sensory phenotyping, when combined with neurobiological and psychophysical methods, might have the potential to provide a better understanding of the sensory processing in ASD and in other NDD.

## Introduction

Sensory difficulties are particularly prevalent in Autism Spectrum Disorder (ASD), affecting 82–97% of individuals [1–4]. Despite Kanner’s early report of sensory atypicalities in ASD and frequent references to sensory abnormalities in autism literature throughout the decades [1, 5], it was not until the 5th edition of the Diagnostic and Statistical Manual of Mental Disorders that sensory symptoms were added to ASD diagnostic criteria [6]. This inclusion triggered an exponential increase in research in the sensory domain in ASD. While the main focus of research on sensory processing disorders has mostly been on adults and children with ASD, there is also a burgeoning sensory-focused interest in other neurodevelopmental disorders (NDD) with or without identified genetic etiologies, such as ADHD [7, 8] Down’s Syndrome [9], Williams Syndrome [10], Fragile X Syndrome [11, 12], Fetal Alcohol Spectrum Disorders [13] and developmental delay [14].

Sensory systems play a central role in the early stages of normal development. They allow us to acquire information from the surrounding world and help us to adapt our behavior to environmental demands [15–17]. Dysfunction in these processes is frequent in NDD and can lead to maladaptive developmental trajectories of cascading delays and deficits with significant impact on development [16, 18–20].

The “sensory symptoms” [21] that are frequently reported by caregivers and systematically assessed in clinical and research settings refer to observable behaviors that occur in response to sensory stimuli. These are frequently classified into patterns of sensory modulation: hypo-responsiveness, which refers to delayed responses or unresponsiveness to sensory stimuli; hyper-responsiveness, which is an exaggerated or even aversive reaction to sensory stimuli; and sensory seeking, which refers to unusual fascination with craving of sensory stimulation, often repetitive in nature [2, 4, 22–25]. Patterns of hyper-, hypo-responsiveness and seeking have high levels of co-occurrence in ASD and other NDD [2, 8], making the comparison between different clinical groups inconsistent. In some studies, hypo-responsiveness distinguishes ASD from other clinical groups [24], while in others, it is hyper-responsiveness [2]. However, using a sensory modulation framework to classify emotional and behavioral responses to sensory stimuli has proven to be useful, especially in the scope of clinical evaluation and intervention [26–28].

In general, neuroscience and psychophysical research in sensory processing use specific experimental paradigms to address unisensory or multisensory processing across modalities (e.g., visual, auditory, tactile, taste and smell) [15, 16, 21]. While these methods allow for precise measurements of neural and psychophysical responses to sensory stimuli, the experimental conditions in which they occur lack ecological validity: they do not reflect the functional demands that arise in daily life, and they do not capture the behavior that arises as an adaptive response to these demands [21]. Parent or proxy-report questionnaires, on the other hand, are among the most widely used assessment methods for sensory symptoms. While they are subject to respondent/caregiver bias, they allow for capturing observed behavior and adaptive efforts to sensory stimuli that occur across different times and contexts. Structured observational protocols, although restrictive in contextual validity, provide opportunities to evaluate observed responses to sensory stimuli in controlled settings and using standardized criteria to classify behavior [29].

Several studies have shown some consistency in the description of the sensory modalities, or sensory systems, that differentiate NDD from each other. For instance, modalities such as audition, taste–smell and touch seem to discriminate individuals with ASD from other clinical groups [4, 7, 14, 30, 31]. Using a modality-based approach to study sensory symptoms could contribute to better delineating specific sensory profiles in ASD and other NDD [4, 7, 14, 30, 31]. Since the implementation of DSM-5, it is widely accepted that “the diagnosis of ASD is a purely behavioral description of a constellation of symptoms” [32, p. 61), which can then be further specified according to the genetic conditions that may accompany the behavioral diagnosis. This constellation of symptoms is highly variable in presence, intensity and presentation across individuals, contributing to the widely acknowledged phenotypic heterogeneity in ASD [33]. This phenotypic heterogeneity in clinical cohorts often hampers our understanding and prevents us from drawing firm conclusions about underlying neurobiological processes.

Groups of individuals sharing the same genetic predisposing factor for NDD offer a unique opportunity to study more homogeneous cohorts and underpin more specific sensory profiles. Copy number variants (CNVs) are microdeletions or microduplications of DNA segments that contribute to inter-individual phenotypic variation [34, 35]. While some are benign, others contribute to a spectrum of NDD and psychiatric disorders, such as developmental delay/intellectual disability (DD/ID), ASD and schizophrenia [36, 37]. Microdeletion at the 16p11.2 locus [29.6–30.2, Hg19] is among the most frequent genetic predisposing factors for NDD and ASD [38–40]. Clinical and cognitive phenotypes of 16p11.2 CNV deletion carriers include obesity, macrocephaly, decreased cognitive functions, as well as speech and language deficits [40–42]. Structural magnetic imaging has shown that 16p11.2 CNV deletion carriers present greater gray matter volume in cortico-subcortical regions implicated in the reward system, language and areas common to idiopathic ASD, including the superior temporal gyrus [43, 44]. The superior temporal gyrus is implicated in the generation of an evoked field component occurring in response to an auditory stimulus and which shows a prolonged latency in 16p11.2 deletion carriers [45]. 16p11.2 deletion carriers also present visual evoked potentials with increased amplitudes [46]. Finally, 16p11.2 heterozygous deletion mice have also shown sensory deficits, notably the absence of startle response due to deafness and increased pain threshold, as well as increased motor stereotypies [47, 48]. While these studies suggest that 16p11.2 deletion carriers present sensory processing vulnerabilities, there are presently no studies investigating the sensory phenotypes of these individuals.

It has been suggested that dysfunctions in sensory processing contribute to the emergence of a diversity of clinical symptoms in ASD and other NDD through cascading effects on developmental acquisitions [49–52]. In ASD, this includes delays in social and communication skills [16, 53–56], maladaptive behaviors [3, 57–59], repetitive behaviors [23, 60–62], and increased anxiety [61–65]. Associations between sensory processing difficulties and clinical symptoms have also been explored across NDD besides ASD, including ADHD [8], Williams Syndrome [66, 67] and other developmental disabilities [16, 56]. This type of approach has the potential to decipher shared and distinct developmental pathways in the context of high comorbidity across disorders.

The goal of this study is to assess sensory processing profiles in three cohorts: children with idiopathic ASD (diagnosis based on clinical phenotype) children, 16p11.2 deletion carriers (genetically driven diagnosis) and typically developing (TD) children. To achieve this, we use a parent-report questionnaire to capture behavioral responses to sensory stimuli across sensory modalities in a straightforward manner. Additionally, we use a laboratory-based observational tactile task to analyze the differences in behavioral responses between the groups. Finally, we explore the associations between sensory processing and different aspects of autistic symptomatology.

## Methods

### Participants

This study includes 121 children diagnosed with Autism Spectrum Disorder (ASD), 17 children carrying a deletion at the 16p11.2 locus (del16p11.2), and 45 typically developing (TD) children. All participants were aged from 2 to 12 years.

#### ASD cohort

All children included in the ASD cohort were patients referred to the Service des Troubles du Spectre de l’Autisme et apparentés at Lausanne University Hospital, Switzerland (STSA-a, CHUV). Formal diagnosis of ASD was established based on the criteria of the Diagnostic and Statistical Manual of Mental Disorders, 5th edition (DSM-5; [6]). Clinical procedure for ASD diagnosis included a review of patients’ medical and developmental history as well as the assessment with the Autism Diagnostic Interview–revised (ADI-R; [68]), and the Autism Diagnosis Observation Scale-2 (ADOS-2; [69]). Trained licensed psychologists and psychiatrists established the ASD diagnosis in a clinical context.

#### 16p11.2 CNV cohort

Carriers of a proximal recurrent 600-kb deletion at the 16p11.2 locus (BP4-BP5; 29.6–30.2 Mb–Hg19) were recruited as part of a larger project on copy number variants and neurodevelopmental disorders. Participants were referred by their clinical geneticist who had initially established the presence of the 16p11.2 microdeletion diagnosis in the context of a neurodevelopmental disorder. Licensed psychologists took a thorough developmental history and performed an ADOS-2 in order to identify the ASD children within this cohort (10/17).

#### Typically developing cohort

TD children were recruited in the general population through distribution of flyers to schools and pediatricians. Exclusion criteria included prematurity (< 36 weeks of gestation), known neurologic (e.g., traumatic brain injury) or neurodevelopmental disorders, a first-degree relative diagnosed with ASD.

### Material

#### Sensory processing questionnaire

The Sensory Processing Measure (SPM; [28, 70]) and the Sensory Processing Measure—Preschool (SPM-P; [27, 71]) are parent-report questionnaires covering a range of behaviors and characteristics related to sensory processing, social participation and praxis. The SPM-P items derive directly from those in the SPM, differing only on a few age-appropriate items. The original SPM consists of three forms that evaluate the child’s functioning in different contexts: Home Form (reported by parents or caregivers), a Main Classroom Form and School Environment Forms. Each form can be used separately.

The age range for SPM-P and SPM was 2–5 and 5–12 years old, respectively. Children aged between 5 and 5 years 11 months were assessed with the SPM if they were attending school. SPM was used for a sample of 52 ASD, 13 del16p11.2 and 31 TD. SPM-P was used for 69 ASD individuals, 4 del16p11.2 and 14 TD were assessed with SPM-P version. Both SPM-P and SPM include 75 items divided into eight subscales: Social Participation (SOC), Planning and Ideas (PLA), Vision (VIS), Hearing (HEA), Touch (TOU), Taste and Smell (TAS), Body Awareness (BOD) and Balance and Motion (BAL). The last two subscales refer to internal sensory modalities—proprioception and vestibular system—, respectively. A raw total Sensory Score (TOT) was calculated based on the raw score of six sensory system subscales (VIS, HEA, TOU, TAS, BOD and BAL). SOC and PLA refer to higher integrative functions (social functioning and praxis, respectively) and hence did not contribute to the TOT score.

Ratings on each item were given on a 4-point Likert scale: 1 (Never), 2 (Sometimes), 3 (Often) and 4 (Always). Raw scores for each subscale were computed by adding the ratings of the subscale’s items. Both SPM-P and SPM present high internal consistency values [27, 28]. In order to merge the scores from both forms, we divided the raw score for each form’s subscale by the number of items included in the subscale, yielding a score from 1 to 4 score for each subscale, as well as for the total score. These computed scores were used as outcome measures.

Hyper-, hypo-responsiveness and seeking global pattern scores were also used in this study. They were computed as the weighted average of the items referred for each pattern across four sensory modalities (e.g., visual, hearing, touch and taste/smell).

#### Tactile defensiveness

The Tactile Defensiveness and Discrimination Test-Revised (TDDT-R; [72, 73]) is a laboratory-based behavioral assessment of tactile processing. This research battery lasts approximately 20 min and was developed for children with developmental difficulties over the age of 3, but is also appropriate to use with TD children [52]. TDDT-R consists of five subtests (*Fuzzy puppet, Sticker game, Treasure hunt Parts 1 and 2 and Feely and Gooey games*) presented in a game-like fashion offering active and passive tactile experiences. Some stimuli are administered by the experimenter (external control items) on the upper part of the child’s body (e.g. q-tips, sticker); while other subtests allow the child to freely explore the stimuli (internal control items) such as sand, play dough and vibrating toys. A tactile defensiveness score ranging from 0 to 3 is derived from the child’s behaviors in response to the stimuli. For the external control items, defensive responses included avoidance or negative affective reactions such as rubbing the skin of the stimulated area, grimacing or negative vocalizations after interaction with the stimulus. For the internal control items, defensive responses included both avoidant behaviors and aversive reactions behaviors to stimuli. Seeking responses were also coded for items in which the child exhibited excessive engagement or a very strong positive affective response to the stimuli. The TDDT-R was administered by trained psychologists, videotaped and scored by consensus between two trained psychologists. The outcome measures used in this study were a) the overall mean tactile defensiveness score obtained by averaging the behavioral responses to internal and external control items and b) seeking score. TDDT-R was performed on 10 ASD participants, on 16 16p11.2 deletion carriers and on 40 TD participants.

#### Overall cognitive functioning

We used two different measures to assess global cognitive development and functioning depending on the child’s age and the ability to comply with the test. The Wechsler Preschool and Primary Scale of Intelligence (WPPSI-IV; [74]) and the Wechsler Intelligence Scale for Children, 5th edition (WISC V; [75]) were used to assess overall cognitive abilities (intelligence quotient, IQ) in children from 2 years 6 months to 12 years of age. Both test batteries included verbal and nonverbal subscales. For the purpose of this study, we used the Nonverbal Intellectual Quotient (NVIQ, mean = 100; standard deviation = 15) as the outcome measure of cognitive level. For younger children and those not being able to comply with the Wechsler scales, we used the Mullen Scales of Early Learning [76]. The MSEL is a measure of cognitive ability and motor development in early childhood (from birth to 5 years 8 months). It comprises 4 subscales: *Gross motor, Fine motor, Visual reception, Receptive language and Expressive language*. For the purpose of this study, we used the Visual Reception (Standard Score) as the outcome measure for global nonverbal abilities. When children fell in the overlapping age ranges between the instruments (mostly in the ASD cohort), the instrument most appropriate to the child’s functioning level was administered in the scope of their clinical evaluation.

In the ASD group, 39 individuals were evaluated with MSEL, 31 with WISC-V and 35 with WPPSI-IV. In the del16p11.2 cohort, 7 deletion carriers were evaluated with WISC-V and 8 with WPPSI-IV. Finally, 21 TD were assessed with WISC-V and 23 with WPPSI-IV.

#### ADOS-2

The Autism Diagnostic Observation Schedule (ADOS-2; [69]) is a semi-structured observational assessment of ASD symptomatology. The ADOS-2 quantifies ASD symptoms in social reciprocity, communication, play and repetitive behaviors. It comprises five modules based on age and language ability (Toddler Module and Modules 1–4). The ADOS-2 adopts an algorithmic scheme, with scores ranging from 0 to 2 for the items with greatest discriminating power being combined into two summary scores: Social Affect (SA) and Restricted and Repetitive Behavior (RRB). Given that a different number of items compose the algorithm across the different modules, we calculated a mean score for the two dimensions (SA and RRB) by dividing the total score in each dimension by the number of items it comprises. We thus obtained a comparable symptom index for SA and RRB across the different modules.

### Statistical analysis

All statistical tests were performed using R 3.6.2 [77]. We performed a multivariate analysis of variance (MANCOVA) to test for group differences using the computed SPM subscale and total scores, controlling for age and gender. When the group main effect was significant, we conducted post hoc analysis. The significance threshold for multiple comparisons was set to 0.017 (0.05/3). As carrying a 16p11.2 deletion is a genetic predisposing factor for ASD, we also compared SPM scores between carriers diagnosed with ASD (*n* = 10) and those without (*n* = 7) using a Mann–Whitney *U* test.

We explored the contribution of each subscale to the total SPM score in the three groups. To do so, we divided VIS, HEA, TOU, TAS, BOD and BAL raw scores by the SPM total raw score, generating a salience value for each sensory domain below or above 1. Scores above 1 indicated that the subscale had a higher contribution to the total score, suggesting that sensory issues in this domain could be more predominant than those on the other subscales. Conversely, scores below 1 indicated a lower contribution of the subscale to the total score. For each sensory modality, we normalized salience values of ASD and 16p11.2 cohorts relative to the salience in the TD group (mean = 0, SD = 1). We used MANCOVA to test for group differences on the normalized salience scores for each sensory domain, with age and gender as covariates.

Finally, we used linear regression to quantify the strength of the linear relationship between the SPM total score and several clinical features. We quantified the regression slope to obtain the estimated effect of a 1-unit increase in SPM total score on NVIQ, ADOS-2 SA, ADOS-2 RBB and ADOS-2 Total scores. The model was applied separately for each clinical group. The two clinical groups were pooled together to test the effect on TDDT-R scores due to insufficient power when separated. We tested the correlation between SPM-touch and TDDT-R defensiveness and seeking. All models used adjusted scores for age and gender.

## Results

Demographics and clinical scores are presented in Table 1. Age was not different between TD and the two clinical cohorts, but ASD individuals were on average one year younger than 16p11.2 deletion carriers (*p* = 0.03). Group-wise Chi-squared tests showed a higher prevalence of males in the ASD (*p* = 3.1e−7) and 16p11.2 (*p* = 0.049) cohorts compared to TD. ASD and 16p11.2 deletion carriers had significantly lower FSIQ compared to TDs (*p* = 4.7e.23 and *p* = 8.9e−9, respectively). Similar results were found for verbal and nonverbal IQ, together with a significant 10-points higher VIQ in del16p11.2 compared to ASD. ASD children did not differ from del16p11.2 deletion carriers in the RRB ADOS-2 scale (*p* = 0.21). However, ASD scored higher in the Social Affect domain (*p* = 5e−4), and ADOS-2 total score (*p* = 4e−4). Tactile defensiveness score, as measured by the TDDT-R, was higher in the ASD and del16p11.2 cohorts compared to TD (*p* < 0.03 for both comparisons). ASD also scored significantly higher than del16p11.2 (*p* = 3.6e−5) and TD (*p* = 6.2e−8) on the TDDT-R seeking scale.

**Table 1:** Sample demographics and clinical phenotype

N	ASD	16p11.2 deletion	TD	Group differences
	121	17	45	–
Mean age (SD)	5.49 (2.9)	6.48 (1.5)	5.85 (2.0)	ASD versus 16p11.2*
Gender (F/M)	18/103	4/13	25/20	ASD versus TD***
				16p11.2 versus TD*
ASD/non ASD	121/0	10/7	0/45	–
Full scale IQ (SD)	79.4 (24.3) (*n* = 105)	86.33 (11.6) (*n* = 15)	115.70 (12.7) (*n* = 44)	ASD versus TD***
				16p11.2 versus TD***
Verbal IQ (SD)	80.1 (24.7) (*n* = 104)	90.3 (10.8) (*n* = 15)	116.6 (12.9) (*n* = 44)	ASD versus TD***
				16p11.2 versus TD***
				ASD versus 16p11.2**
Nonverbal IQ (SD)	86.7 (22.6) (*n* = 105)	92.1 (12.1) (*n* = 17)	112.6 (12.4) (*n* = 44)	ASD versus TD***
				16p11.2 versus TD***
ADOS SA(SD)	1.04 (0.3) (*n* = 112)	0.68 (0.4) (*n* = 16)	–	ASD versus 16p11.2***
ADOS RRB (SD)	0.94 (0.4) (*n* = 111)	0.71 (0.6) (*n* = 16)	–	
ADOS total (SD)	1.01 (0.3) (*n* = 111)	0.69 (0.4) (*n* = 16)	–	ASD versus 16p11.2***
TDDT defensiveness (SD)	0.29 (0.4) (*n* = 12)	0.31 (0.4) (*n* = 16)	0.06 (0.1) (*n* = 40)	ASD versus TD*
				16p11.2 versus TD**
TDDT SSK (SD)	0.16 (0.2) (*n* = 14)	0.01 (0.0) (*n* = 16)	0.00 (0.0) (*n* = 40)	ASD versus TD***
				ASD versus 16p11.2***

*ASD* Autism Spectrum Disorder, *IQ *Intelligence Quotient*, ADOS-2 SA* ADOS-2 Social Affect, *ADOS RRB* ADOS Restricted and Repetitive Behaviors, *TDDT* Tactile Defensiveness and Discrimination Test—Revised, *SSK* Seeking, *SD* Standard Deviation

^*^p < 0.05; **p < .0.01; ***p < 0.001

**Fig. 1:**
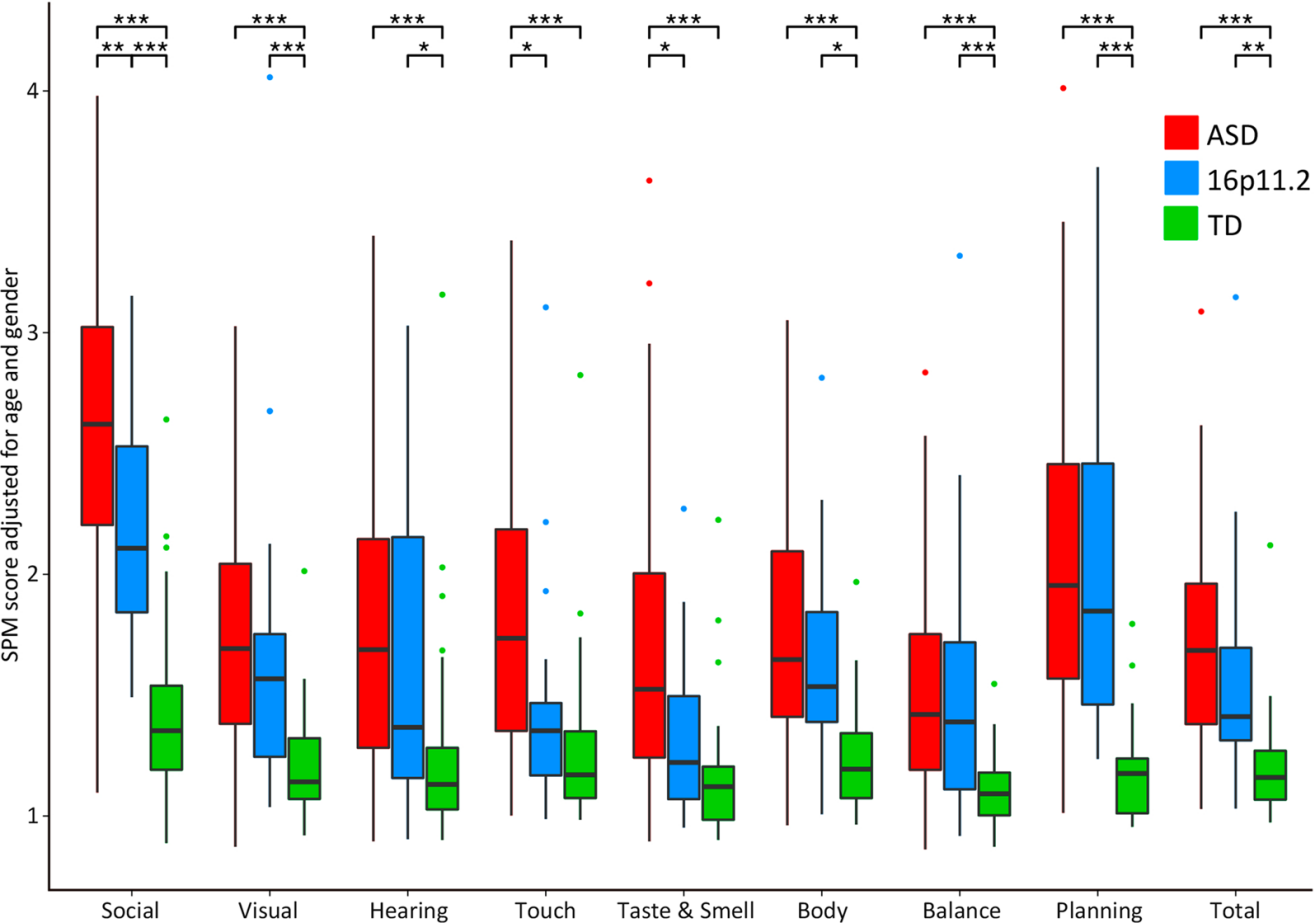
Group comparison on the SPM mean raw scores, adjusted for age and gender. Sensory scores as a function of modality and group (ASD, 16p11.2 deletion carriers and TD). Boxplots represent the SPM raw scores adjusted for age and gender. The bold black line inside each boxplot shows the median, and the bottom and top of the box show the 25th (quartile 1 [Q1]) and the 75th (quartile 3 [Q3]) percentile, respectively. The upper whisker ends at highest observed data value within the span from Q3 to Q3 + 1.5 times the interquartile range (Q3–Q1), and lower whisker ends at lowest observed data value within the span for Q1 to Q1-(1.5 * interquartile range). Points not reached by the whiskers are outliers. Significant post hoc group differences are labeled with stars on the top of the figure (*p < 0.05, **p < 0.01 and ***p < 0.001 after Bonferroni correction)

The MANCOVA showed a significant group effect on the multivariate SPM score (*p* = 1.1e−17). ASD children scored significantly higher on all SPM subscales compared to TD, particularly on the Social Communication subscale (Fig. 1). 16p11.2 deletion carriers also scored higher than TD on all subscales, except for touch and taste/smell. Touch and taste/smell were also the two sensory domains that yielded significant differences between the ASD and 16p11.2 cohorts (*p* = 0.01 and *p* = 0.009, respectively). These results were independent of the overall nonverbal functioning. When adding the NVIQ as a covariate in the analysis, differences in touch and taste/smell scores between ASD and 16p11.2 individuals remained statistically significant (*p* = 0.0159 and *p* = 0.0153, respectively). All scores and p-values are reported in Additional file 1: Table S1. The MANCOVA showed an overall significant age and gender effect across groups (*p* = 2.8e−10 and *p* = 0.021, respectively). SPM raw scores were systematically higher in males than females. We also observed higher scores in younger individuals.

We also compared 16p11.2 CNV deletion carriers with and without an ASD diagnosis (Additional file 1: Table S2). Del16p11.2 diagnosed with ASD scored higher on the body (*p* = 0.007) and planning (*p* = 0.019) subscales, compared to 16p11.2 non-ASD carriers. These two subgroups showed similar scores in the rest of the subscales as well as on the TDDT-R Defensiveness and Seeking measures.

**Fig. 2:**
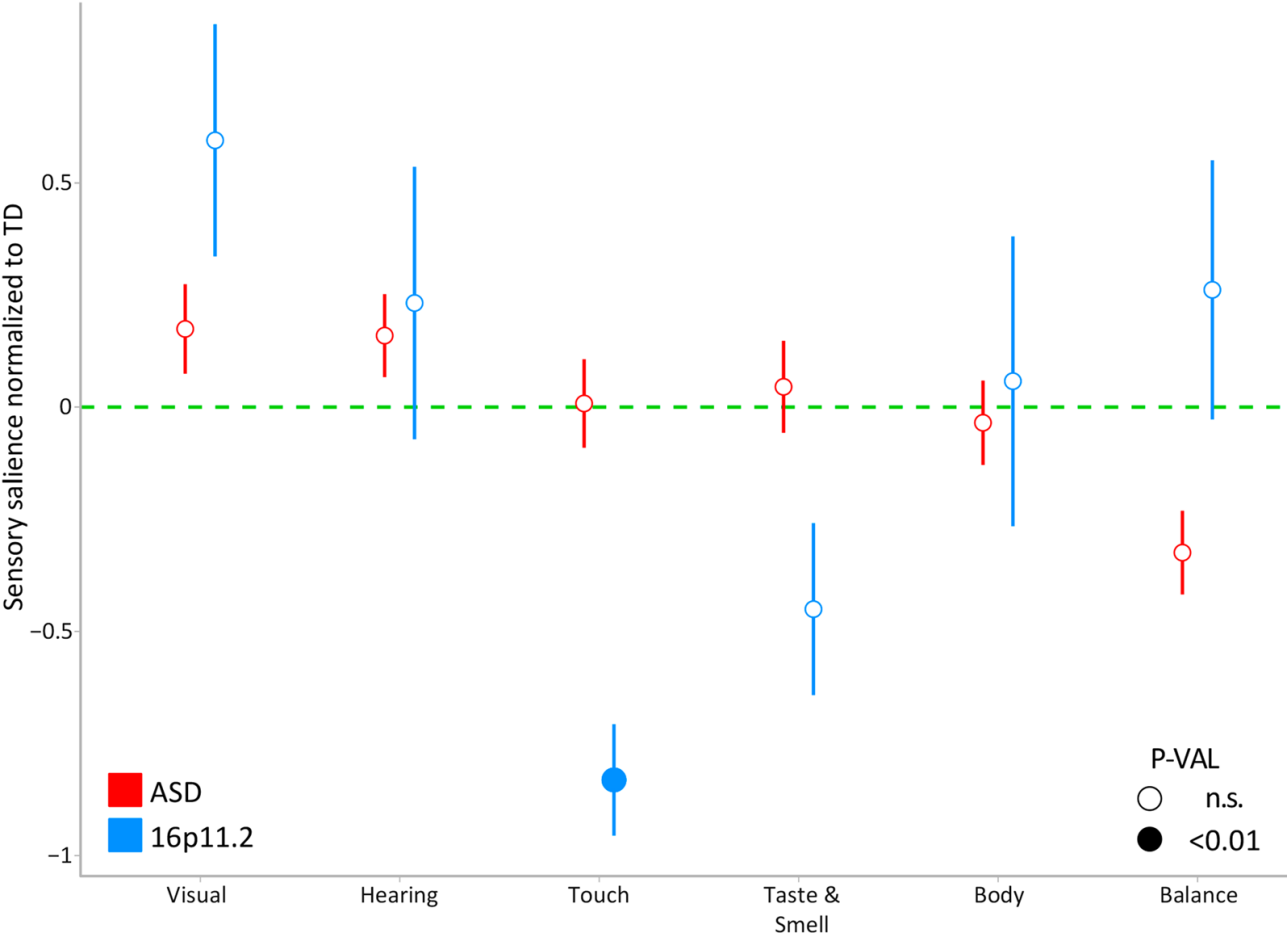
Salience of SPM scores. Salience score of the SPM adjusted for age and gender across modalities in both clinical groups (ASD, 16p11.2 deletion carriers). Scores are means normalized to TD (green dotted line). Error bars represent the standard error of the mean

To assess the contribution of each SPM subscale to the total score, we computed a salience score, defined as the ratio between the subscale and the SPM total (see “Methods” section). The MANCOVA highlighted a significant group effect (*p* = 0.017) in the salience scores after accounting for age and gender effects (Fig. 2). Post hoc tests showed that touch salience was significantly lower in del16p11.2 deletion carriers compared to ASD (*p* = 0.001) and TD (*p* = 0.004).

The contribution of the Taste/Smell subscale to the total score in 16p11.2 deletion carriers was also below that of TD, but did not reach statistical significance. No group differences were found regarding other score saliences in other sensory domains. All other subscales presented an average salience above TD, suggesting that behavioral responses to touch and taste/smell stimuli might reflect less difficulties in 16p11.2 deletion carriers, as opposed to the idiopathic ASD cohort. The contribution of each sensory modality in the ASD cohort is not different from TD, which is consistent with the pattern observed in Fig. 1.

**Fig. 3:**
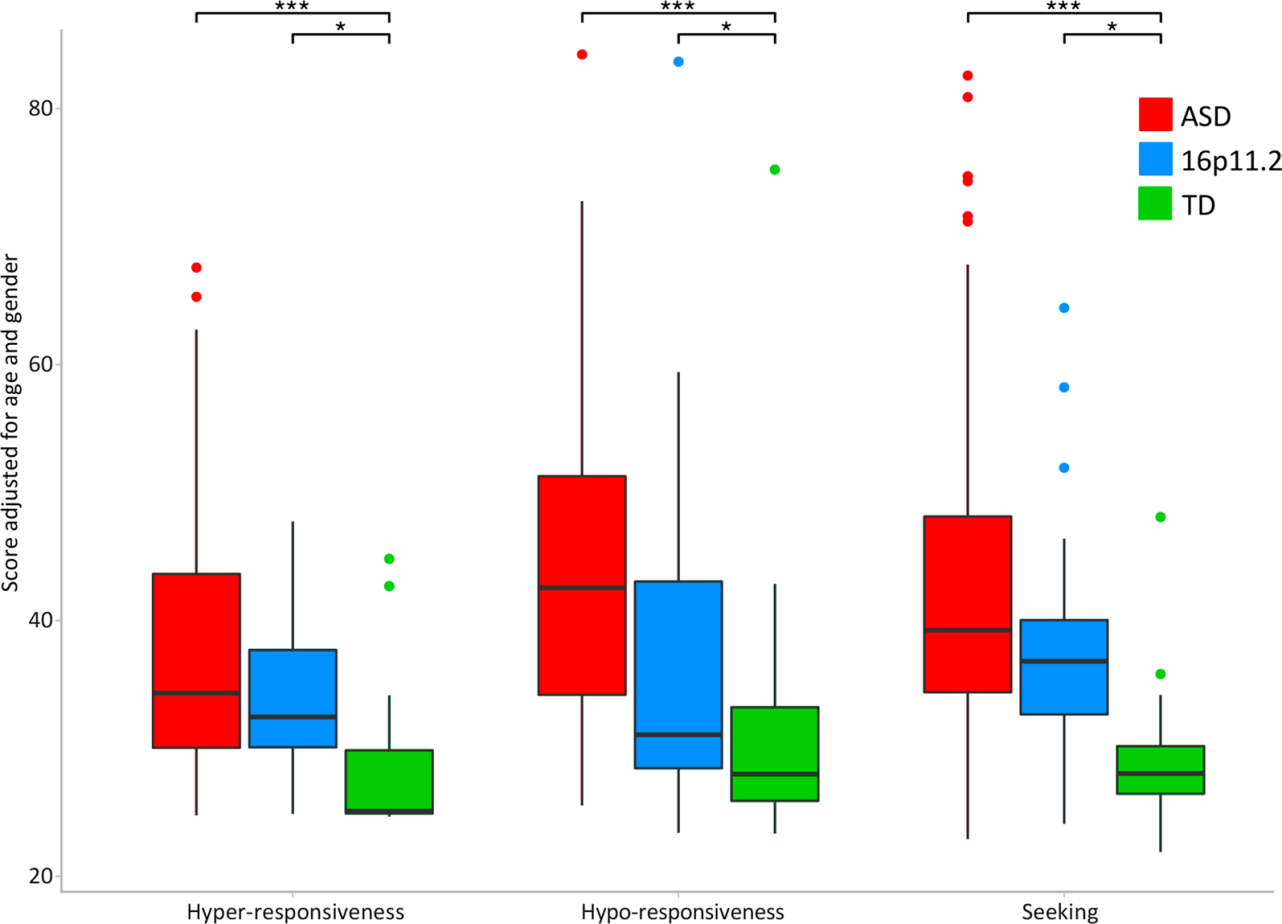
Group comparison on modulation patterns. Sensory scores as a function of the modulation pattern of response (hyper-, hypo-responsiveness, and seeking) and group (ASD, 16p11.2 deletion carriers and TD). Boxplots represent the SPM raw scores adjusted for age and gender. The bold black line inside each boxplot shows the median, and the bottom and top of the box show the 25th (quartile 1 [Q1]) and the 75th (quartile 3 [Q3]) percentile, respectively. The upper whisker ends at highest observed data value within the span from Q3 to Q3 + 1.5 times the interquartile range (Q3–Q1), and lower whisker ends at lowest observed data value within the span for Q1 to Q1-(1.5 * interquartile range). Points not reached by the whiskers are outliers. Significant post hoc group differences are labeled with stars on the top of the figure (*p < 0.05, **p < 0.01 and ***p < 0.001 after Bonferroni correction)

Group comparison based on the modulation pattern of responses (hyper, hypo, sensory seeking) did not differentiate the del16p11.2 and ASD cohorts in any of the patterns (*p* > 0.1 for all comparisons, Fig. 3). However, both groups scored significantly higher than TD in all three response patterns (*p* < 0.012 for all comparisons).

**Fig. 4:**
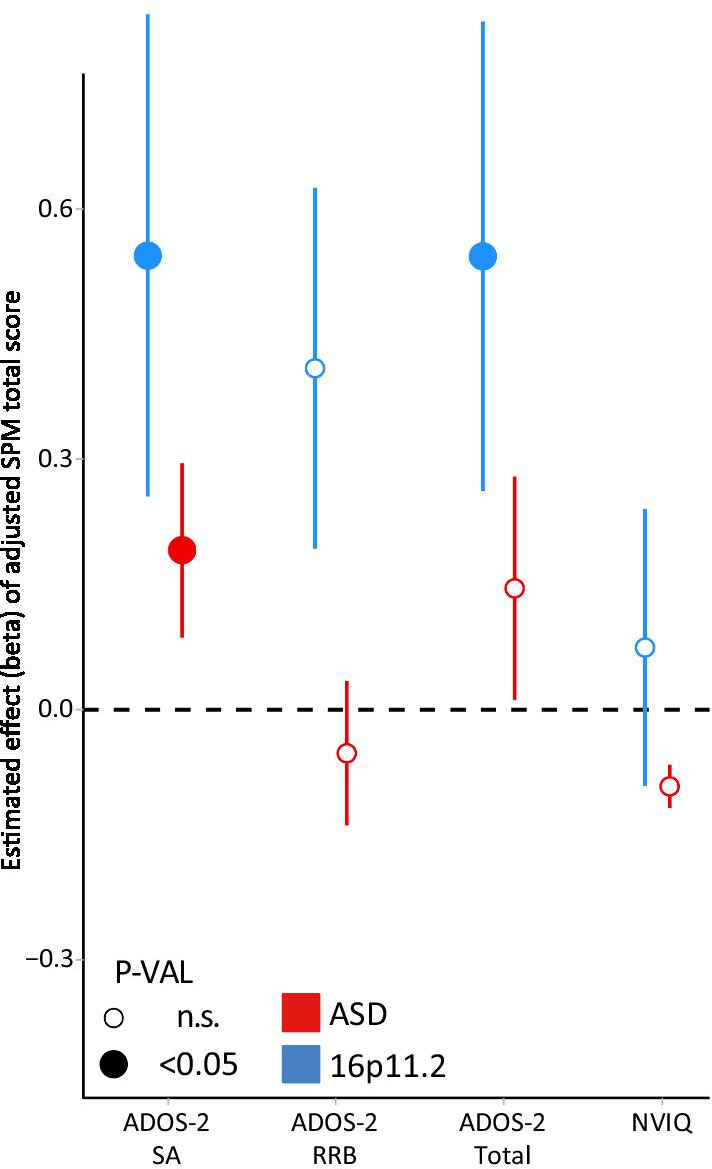
Association between SPM total score and other continuous phenotypes. Estimated change (regression slope) in the expected value of several phenotypes for a given value of SPM total score. Y-axis values are the effect of a 1-unit increase in SPM total score in the expected value of NVIQ, ADOS-2 SA, ADOS-2 RBB and ADOS-2 Total scores, separately for ASD (red) and 16p11.2 deletion carriers (blue). Error bars represent standard error. The significance threshold was set at *p* < 0.05

Finally, we performed linear regressions to analyze the association between sensory SPM scores and direct observation measures such as ADOS-2 dimensions of Social Affect (SA), Restrictive and Repetitive Behavior (RRB) as well as total ADOS-2 score and NVIQ (Fig. 4; Additional file 1: Fig. S2). We found a positive relationship between the SPM total score and the ADOS-2 SA scale in both the ASD and the del16p11.2 cohort (*p* = 0.04 and *p* = 0.03, respectively), but not with the RRB. We also found a positive relationship between SPM total score and ADOS-2 total score, but only in the del16p11.2 group (*p* = 0.03). There was no significant association with NVIQ. We found no significant association between the SPM touch score and neither TDDT-R defensiveness nor seeking scores (Additional file 1: Fig. S1).

## Discussion

The present study used a symptom-based approach to investigate sensory atypicalities in two clinical groups: a cohort of children with ASD and children carrying a microdeletion at the 16p.11.2 locus. This study is also the first to describe the sensory phenotype of 16p11.2 deletion carriers.

Our main finding shows that tactile and olfactory/taste processing are the only two domains that differentiate young carriers of a 16p11.2 deletion from ASD children. While ASD children have more sensory atypicalities across all modalities compared to TD, which is consistent with prior studies [2, 4, 55, 78], 16p11.2 deletion carriers score higher on all subscales except in the tactile and olfactory/taste domains.

These results suggest that behavioral responses to sensory stimuli in olfaction/taste and tactile domains, as measured by parent-report, are specifically affected in ASD and contribute significantly to the phenotype of this disorder. Atypical responses to touch and olfaction/taste are indeed particularly salient in the daily life of ASD children and adults, as reported by parents [1, 79–81], and in individual or anecdotal accounts [82–84]. Children are frequently reported to react emotionally to being touched by other people, becoming irritated by having tags on their clothes, having decreased responses to pain and temperature or displaying an unusual need for touching or avoiding certain surfaces or textures. They can also display narrower food preferences, an unusual tendency to explore objects or people by smelling them or strong preferences for certain smells and taste [27, 28, 78].

We used a laboratory-based observational instrument to evaluate tactile defensiveness and seeking (TDDT-R; [72, 73]). We did not find a significant association between the scores on the touch domain of the SPM and the TDDT-R. Additionally, the results obtained with the TDDT-R showed that 16p11.2 participants had significantly higher levels of tactile defensiveness than ASD children, contrary to the results obtained from the parent-report measure. This is likely due to the nature of the process that was assessed. The touch subscales of the SPM and SPM-P encompass different responses to various types of tactile stimuli, including response to light touch, pain, as well as self-directed tactile exploration, and tactile discrimination [70, 71]. In the TDDT-R, however, the only measure adopted was tactile defensiveness, conceptualized as negative and emotional responses to touch [72, 73]. These results highlight the methodological challenges in capturing ecologically valid laboratory-based observable behaviors that allow researchers to delve further into the mechanisms that underlie observable, everyday functioning. As such, our results also highlight the need to fine-tune self-report measures in order to capture real-life correlates of the behaviors represented in the TDDT-R. Finally, only a small subset of ASD individuals was assessed with TDDT-R, as only ASD children taking part in a larger research project were administered the task. A larger sample size and a finer grained analysis of tactile processing in ASD and 16p11.2 would ultimately allow us to better understand tactile processing difficulties and to what extent these particularities are specific to ASD, or if they are also present in different degrees across NDD.

In the tactile domain, recent work [17, 85] suggests that “social touch” is altered in ASD. This term refers to the activation of specific tactile fibers that respond preferentially to the gentle, caress-like stroking that is characteristic of inter-individual touch in relationships and social contexts [17]. ASD individuals would show more defensive reactions to social touch compared to regions associated with palmar touch that is typically used for discrimination [85]. Clinical observations and anecdotal reports from ASD individuals often mention difficulties in tolerating other-initiated light touching, while seeking self-initiated tactile stimulations such as pressure [86]. However, this differential preference for tactile stimuli is not captured in detail by the available parent-report instruments. The interdisciplinary combination of behavioral measures with self-report questionnaires, and this with further neurophysiological testing, remains a promising avenue for further understanding sensory processing and its underlying mechanisms.

A recent meta-analysis confirmed that olfactory dysfunction might be strongly associated with ASD [87]. ASD children and adults seem to exhibit normal odor detection, while the identification of odors seems to be more impaired than in typically developing participants [88].

Even though we found differences across sensory modalities, we were unable to differentiate ASD and 16p11.2 groups when using a modulation pattern approach (hyper-, hypo-responsiveness and seeking). Findings in the literature have been inconsistent when attempting to find patterns of sensory modulation that differentiate ASD from other NDD: while some studies consider that hypo-responsiveness could be the distinguishing feature between children with ASD and those with other developmental delays [24], others consider that it is hyper-responsiveness[2]. This can be due to the fact that behavioral responses to sensory stimuli do not necessarily reflect underlying mechanisms. For example, although it might be tempting to assume that hyper-responsive individuals have a low threshold and require less sensory input to generate a typical response, hyper-responsiveness could also be due to other mechanisms such as impaired habituation [20, 21]. The inconsistencies in finding patterns that distinguish ASD from other NDD can also be due to a co-occurrence of sensory patterns, even within one modality [20, 31].

We found a significant age effect on the SPM across all three groups, suggesting a decrease in atypicalities as children grow older. These results are consistent with what is observed in typically developing children [14, 27, 28]. The results from studies looking at ASD children are less consistent, although the majority points to a similar trend [7, 78, 89, 90]. We also found a gender effect suggesting more atypical responses to sensory stimuli in males relative to females. The literature looking at the effect of gender in sensory processing in the ASD population is limited, especially due to the imbalance of the female to male ratio in ASD samples [2]. However, these observations seem to adopt the same trend as in typically developing children. Indeed, the validation study of the SPM and SPM-P showed an overall gender effect and reported that although the effect size was small and the results were clinically insignificant, male children had higher scores in the SPM and SPM-P scales [27, 28]. Research on gender differences in sensory processing is scarce and should be explored in future research.

Studies that have focused specifically on tactile or olfactory/taste processing in ASD yield inconsistent results likely due to methodological differences [20, 91]. Assessment tools are limited in clinical settings and development of innovative approaches to investigate these domains is needed. It is important to keep in mind that sensory processing is a cascade of events that involves sequential steps from the conversion of physical information to electrical information to the conscious and subconscious selection of emotional and behavioral responses [20]. Dysfunction can occur at any step of the chain. Observable reactions do not capture sensory processing as a whole [21]. In this context, combining different methods would help to understand the behavioral responses that occur in environmental settings and the neural and psychophysiological mechanisms that underlie them. As an example of interdisciplinary research, behavioral measures of response to tactile stimuli have shown to be correlated with white matter microstructure in children with and without sensory processing disorders [92]. Using a modality-based approach at all levels (parent-report, observation protocol and neurophysiological measures) could thus be a useful strategy for reducing the discrepancy and allowing cross-method comparisons [1, 30].

Even though clinical reports and research literature have supported an association between touch, olfaction and gustatory issues with ASD and its core symptomatology, the nature of this relationship remains unclear [20, 80, 87]. In the present study, we found that sensory issues were associated with ADOS-2 Social Affect scores in both clinical groups, suggesting that sensory issues may play an important role in social communication processes. In the del16p11.2 cohort, overall sensory dysfunction was also related to ADOS-2 Total, suggesting that 16p11.2 deletion carriers with more sensory issues were more likely to present autistic symptomatology. Further research into the differential relationships between sensory issues and core symptomatology of ASD across different NDD is warranted. This could help shed light on the common clinical features that exist between different disorders, as well as the mechanisms that contribute to their development. Additionally, it would be interesting to investigate how tactile and olfactory/gustatory abnormalities are associated with clinical phenotypes. Initial evidence points to a link between these domains and core ASD symptoms [55, 57, 88, 93, 94].

Although it is widely accepted that sensory issues could present specific profiles across different NDD, a vast majority of the studies that have attempted to compare ASD to other clinical populations have mostly relied on mixed-etiology clinical samples, including individuals with different genetic disorders and idiopathic developmental delay [2]. Furthermore, the widely recognized heterogeneity of idiopathic ASD makes it difficult to pinpoint the sensory processing difficulties that are specific to this disorder [29, 33]. This heterogeneity has important implications at all levels, from addressing the underlying biological mechanisms to tailoring specific interventions to the specific profile of the individual [33]. Nevertheless, there has been an interest in delineating the profiles of sensory processing in more homogenous groups, such as those with certain genetic syndromes [18]. We used a cohort of 16p11.2 deletion carriers and found that they present a distinct profile from idiopathic ASD. Using a “genetic-first” approach as focusing on CNV carriers to study cohorts of patients sharing similar molecular alterations, and in this case, sharing a fairly homogeneous clinical phenotype offers an alternative to the classical research “phenotype-first” methodology classically used in psychiatry research.

### Limitations

Some limitations need to be acknowledged. First, our dataset mostly relies on a parent-report questionnaire, which can be biased by parents’ expectations and judgments. However, the use of questionnaires also has the advantage of ecological validity, which is not possible when using a more experimental approach (e.g., psychophysics, electrophysiology). Even though the SPM questionnaire is not able to capture sensory processing in all its complexity, it allows for a simple and rapid collection of data across modalities. This is useful when comparing sensory response patterns between clinical samples, as is the case in this research with 16p11.2 deletion carriers and ASD children.

Second, the sample size of our 16p11.2 CNV deletion cohort is smaller than the two other cohorts. Since a CNV at this locus is considered rare (~ 1/2000 people) and, in this study, we focus on early childhood, our sample size is limited. That being said, grouping individuals with the same genetic risk factor for ASD (del16p11.2) substantially reduces the heterogeneity of the population, which generally comes with larger effect sizes. Nevertheless, larger datasets of rare genetic rearrangements are still warranted to improve statistical power. We also acknowledge the low number of ASD participants with TDDT-R data affecting the statistical power of the exploratory analysis.

Finally, based on the abovementioned factors, we acknowledge that the differences between the ASD and del16p11.2 could be argued to be exploratory. In this sense, our results warrant corroboration from replication studies.

### Future investigations and clinical implications

Our results open new avenues to the investigation of sensory processing in ASD and other NDD. Although olfaction/taste and tactile processing remain insufficiently investigated, our study and prior literature point to a specificity in theses domains in ASD. The nature of the link between response patterns at the clinical level and the underlying neural and physiological mechanisms remains unclear. It will be important in future studies to combine methods to capture sensory processing at several levels and investigate how these are integrated. Studying different relatively homogeneous genetic cohorts to evaluate sensory responses seems to open promising ways to better understand and to decipher sensory mechanisms.

## Conclusions

Our study highlights the high vulnerability to touch and olfaction/taste stimuli in the environment of ASD children when compared to a cohort of children with 16p11.2 CNV deletion and typically developing children. Further specific research in these sensory domains is warranted to better understand early alteration in ASD brain development. As typically developing individuals, we underestimate how disruptive such stimuli can be in everyday life. A better knowledge of sensory processing in these two domains is the first step to understanding how to adapt the environment and tailor therapies in order to improve children’s behavior and difficulties and, ultimately, their quality of life.

## Supplementary information

**Additional file 1.** Touch and olfaction/taste differentiate children carrying a 16p11.2 deletion and children with ASD.

## Data Availability

The datasets used and/or analyzed during the current study are available from the corresponding author on reasonable request.

## Abbreviations

ASD: Autism spectrum disorder
NDD: Neurodevelopmental disorders
TD: Typically developing
ADOS-2: Autism Diagnostic Observation Schedule—Version 2
SA: ADOS-2 Social Affect
RRB: ADOS-2 Restricted and Repetitive Behaviors
CNV: Copy Number Variant
TDDT-R: Tactile Defensiveness and Discrimination Test-Revised
SPM: Sensory Processing Measure
SPM-P: Sensory Processing Measure Preschool
ADHD: Attention Deficit Hyperactivity Disorder
NVIQ: Nonverbal IQ
FSIQ: Full Scale IQ
