# Supplementary material for: Touch and olfaction/taste differentiate children carrying a 16p11.2 deletion from children with ASD
Source: Mol Autism. 2021 Feb 5;12:8. doi: 10.1186/s13229-020-00410-w (PMC7863523; doi:10.1186/s13229-020-00410-w)
Supplement: Supplementary file 1 — Additional file 1. Touch and olfaction/taste differentiate children carrying a 16p11.2 deletion and children with ASD. [file 13229_2020_410_MOESM1_ESM.docx]

**Touch and olfaction/taste differentiate children carrying a 16p11.2 deletion and children with ASD**

***Supplemental Information***

**Table S1: MANOVA results on SPM mean raw scores and standard deviation**

|  | ASD  Mean (SD) | 16p11.2  Mean (SD) | TD  Mean (SD) | ASD vs 16p  p-value | ASD vs TD  p-value | 16p vs TD  p-value |
| --- | --- | --- | --- | --- | --- | --- |
| Social | 2.58 (0.6) | 2.12 (0.5) | 1.46 (0.4) | **0.003** | **1.7e-22** | **8e-6** |
| Visual | 1.76 (0.5) | 1.72 (0.8) | 1.20 (0.2) | 0.971 | **2e-9** | **4.7e-4** |
| Hearing | 1.78 (0.6) | 1.69 (0.7) | 1.25 (0.4) | 0.488 | **1.3e-7** | **0.003** |
| Touch | 1.81 (0.6) | 1.47 (0.5) | 1.27 (0.3) | **0.012** | **5.2e-8** | 0.148 |
| Smell/Taste | 1.65 (0.5) | 1.32 (0.4) | 1.17 (0.2) | **0.009** | **2.4e-8** | 0.148 |
| Body | 1.77 (0.5) | 1.62 (0.5) | 1.23 (0.2) | 0.21 | **3e-9** | **0.003** |
| Balance | 1.50 (0.4) | 1.57 (0.6) | 1.13 (0.1) | 0.728 | **1.1e-7** | **1.2e-4** |
| Planning | 2.00 (0.6) | 1.99 (0.7) | 1.18 (0.2) | 0.923 | **4.4e-14** | **3.9e-7** |
| Total | 1.72 (0.4) | 1.59 (0.5) | 1.21 (0.2) | 0.203 | **8.4e-12** | **3.7e-4** |

The first three columns report the mean and standard deviation (SD) of the 8 SPM subscales and SPM total for each group (ASD, del16p11.2, and TD). The last three columns report the p-values obtained when doing post-hoc group-wise comparisons using ANCOVAs (age and gender as covariates). The significant threshold was set to alpha = 0.05/3 = 0.017 (Bonferroni correction). Significant group differences are in bold. The group effect on the multivariate mean across scores (MANCOVA with age and gender as covariates) was found to be significant, with p = 1.1e-17. Age and gender also had a significant effect on the multivariate mean (p=2.8e-10 and p=0.021, respectively).

**Table S2: Group comparison on the SPM and TDDT-R mean scores (adjusted for age and gender) between 16p11.2 carriers with and without ASD**

|  | ASD 16p11.2  N= 10  Mean (SD) | Non-ASD 16p11.2  N= 7  Mean (SD) | p-value |
| --- | --- | --- | --- |
| SPM-Social | 2.34 (0.5) | 1.89 (0.3) | 0.088 |
| SPM-Visual | 1.99 (0.9) | 1.43 (0.4) | 0.088 |
| SPM-Hearing | 1.87 (0.7) | 1.4 (0.7) | 0.161 |
| SPM-Touch | 1.62 (0.6) | 1.27 (0.2) | 0.475 |
| SPM-Taste | 1.45 (0.4) | 1.19 (0.3) | 0.27 |
| SPM-Body | 1.84 (0.4) | 1.31 (0.3) | **0.007** |
| SPM-Balance | 1.72 (0.7) | 1.31 (0.3) | 0.315 |
| SPM-Planning | 2.28 (0.6) | 1.60 (0.5) | **0.019** |
| SPM-Total | 1.78 (0.6) | 1.34 (0.2) | 0.109 |
| TDDT-R  Defensiveness | 0.21 (0.2) | 0.44 (0.5) | 0.43 |
| TDDT-R  Seeking | 0 (0) | 0.02 (0.05) | 0.31 |

The first two columns report the mean and standard deviation (SD) of the 8 SPM subscales, the SPM total and TDDT-R Defensiveness and Seeking for 16p11.2 CNV deletion carriers with and without ASD, respectively. The last column reports the p-values obtained from the Mann–Whitney U test after adjusting for age and gender. The significant threshold was set to alpha = 0.05/2 = 0.025 (Bonferroni correction). Significant group differences are in bold.

**Table S3: SPM mean scores (adjusted for age and gender) between idiopathic ASD and 16p11.2 deletion carriers with ASD**

|  | Idiopathic ASD  N= 121  Mean (SD) | 16p11.2 ASD  N= 10  Mean (SD) | p-value |
| --- | --- | --- | --- |
| Social | 2.58 (0.6) | 2.34 (0.5) | 0.172 |
| Visual | 1.76 (0.5) | 1.99 (0.9) | 0.729 |
| Hearing | 1.78 (0.6) | 1.87 (0.7) | 0.768 |
| Touch | 1.81 (0.6) | 1.62 (0.6) | 0.189 |
| Taste | 1.65 (0.5) | 1.45 (0.4) | 0.237 |
| Body | 1.77 (0.5) | 1.84 (0.4) | 0.599 |
| Balance | 1.50 (0.4) | 1.72 (0.7) | 0.717 |
| Planning | 2.00 (0.6) | 2.28 (0.6) | 0.217 |
| Total | 1.72 (0.4) | 1.78 (0.6) | 0.881 |

We used a Mann-Whitney U test to compare the SPM subscale and total scores between idiopathic ASD participants and 16p11.2 carriers with ASD, adjusted for age and gender. The first two columns report the mean and standard deviation of the eight SPM subscales and SPM total for idiopathic ASD and 16p11.2 ASD, respectively. The last column reports the p-values. No significant differences were found.

**Figure S1:**

**
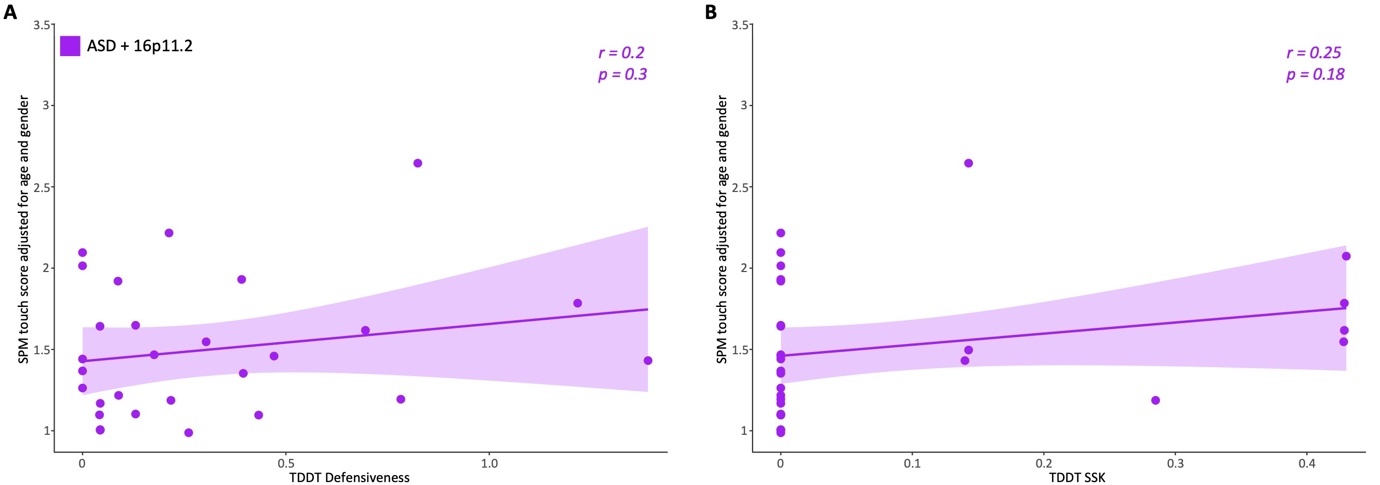
**

**Figure S1.** Scatter plots showing the linear correlation between SPM touch and the **(A)** TDDT-R Defensiveness mean score and **(B)** the TDDT-R Seeking mean score, when the two clinical cohorts (ASD + 16p11.2) are merged. Both panels include the regression line, Pearson’s correlation coefficient, and p-value. Shaded areas depict the 95% confidence intervals of the regression line.

**Figure S2:**

**
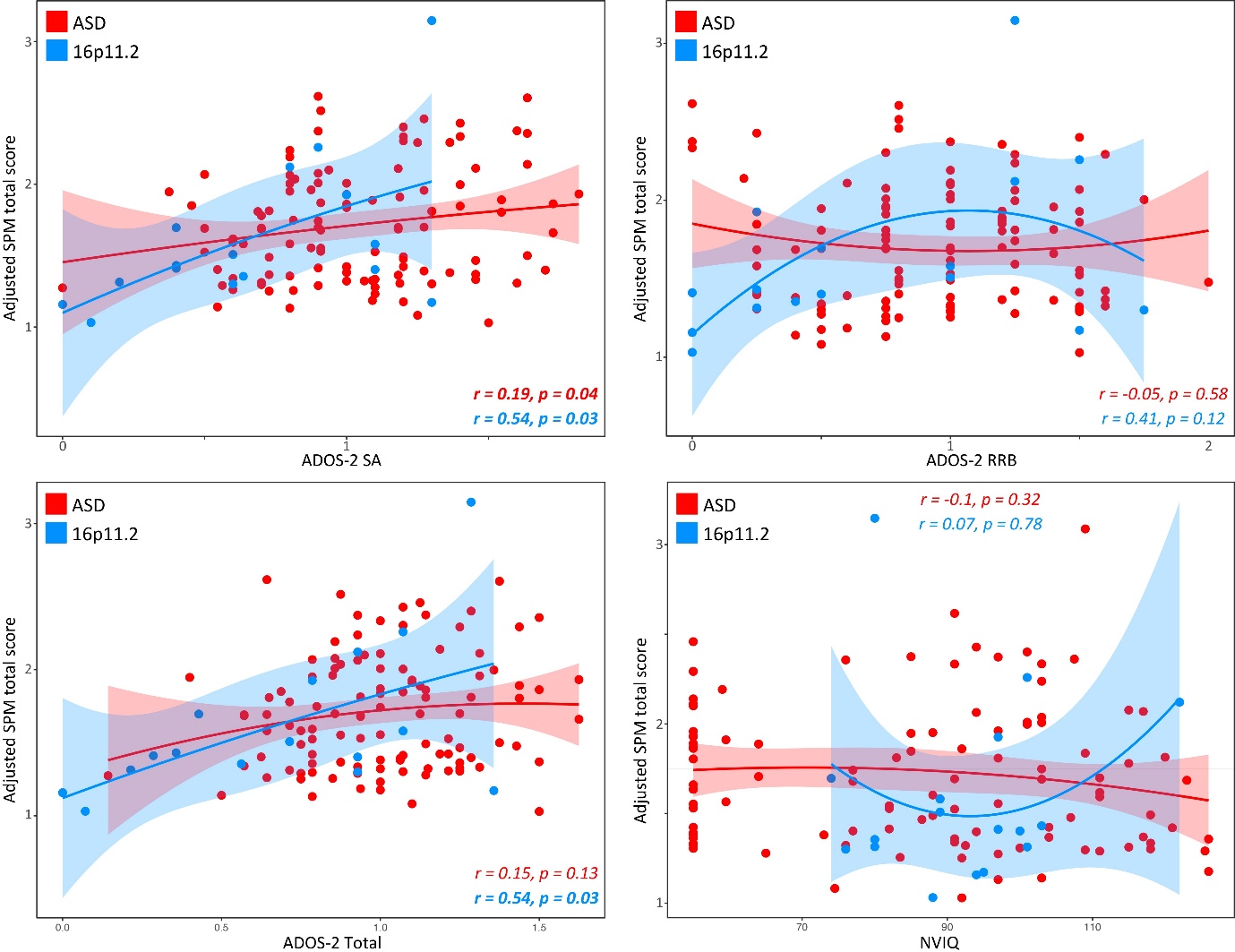
**

**Figure S2.** Scatter plots showing the correlation in ASD (red) and 16p11.2 (blue) cohorts between SPM total score adjusted for age and gender and four direct observation measures: ADOS-2 Social Affect, ADOS-2 Restricted and Repetitive Behavior, ADOS-2 Total and nonverbal IQ. Both panels include the regression line, Pearson’s correlation coefficient, and p-value. Shaded areas depict the 95% confidence intervals of the regression line.
